# Protocol for Nearly Full-Length Sequencing of HIV-1 RNA from Plasma

**DOI:** 10.1371/journal.pone.0001420

**Published:** 2008-01-09

**Authors:** Yuka Nadai, Lindsay M. Eyzaguirre, Niel T. Constantine, Anne M. Sill, Farley Cleghorn, William A. Blattner, Jean K. Carr

**Affiliations:** 1 Department of Epidemiology, Institute of Human Virology, University of Maryland, Baltimore, Maryland, United States of America; 2 Futures Group, Washington, D. C., United States of America; National Cancer Institute, United States of America

## Abstract

Nearly full-length genome sequencing of HIV-1 using peripheral blood mononuclear cells (PBMC) DNA as a template for PCR is now a relatively routine laboratory procedure. However, this has not been the case when using virion RNA as the template and this has made full genome analysis of circulating viruses difficult. Therefore, a well-developed procedure for sequencing of full-length HIV-1 RNA directly from plasma was needed. Plasma from U.S. donors representing a range of viral loads (VL) was used to develop the assay. RNA was extracted from plasma and reverse-transcribed. Two or three overlapping regions were PCR amplified to cover the entire viral genome and sequenced for verification. The success of the procedure was sensitive to VL but was routinely successful for VL greater than 10^5^ and the rate declined in proportion to the VL. While the two-amplicon strategy had an advantage of increasing the possibility of amplifying a single species of HIV-1, the three-amplicon strategy was more successful in amplifying samples with low viral loads. This protocol provides a useful tool for molecular analysis to understand the HIV epidemic and pathogenesis, as well as diagnosis, therapy and future vaccine strategies.

## Introduction

Since the first complete sequence of the HIV-1 genome was published in 1985 [1], there have been numerous reports documenting the genetic variability of the virus based on sequencing. In the HIV sequence database [2] there are currently 1404 HIV-1 sequences greater than 8 kb in length. However, the vast majority of these sequences have been generated using DNA from either peripheral blood mononuclear cells (PBMC) or cultured cells [1], [3]–[5]. In fact, only 74 sequences from 47 individuals are identified as derived from RNA and all of them were generated using cloning; there are no full-length HIV-1 sequences that are clearly from direct PCR amplification and sequencing of virion RNA. The reason for this is that generating a single, nearly full-length PCR amplicon of HIV-1 using virion RNA as the template has been relatively difficult.

The ability to monitor the sequence of the full genome of HIV-1 in the virion is important for many reasons. Virion RNA reflects currently replicating virus, and thus has more pathogenic significance than the proviral DNA present in PBMC [6]–[9]. Proviral DNA contains high levels of integrated defective viral sequences [6]. Thus the analysis of virion RNA is ideal for studying the pathogenesis and epidemiology of HIV-1. Moreover, it is often the case that PBMC are not available from important study populations, and studies must be conducted using plasma or serum. Such studies have often been restricted to partial genome characterization because of the technical difficulty of using RNA as the template [10], [11].

A well-developed strategy for amplification and sequencing of nearly full-length HIV-1 directly from plasma viral RNA was needed. Standard sequencing methods require efficient PCR amplification and this is more difficult in viral RNA samples because of common problems in synthesizing full-length cDNA. Recent improvements in commercially available RNase H^−^ free reverse transcriptase (RT) have made it possible to synthesize cDNA for nearly full-length HIV-1 RNA genomes (∼9 kb). In this report, optimal conditions for reverse transcription and PCR amplification (RT-PCR) of HIV-1 RNA in plasma samples were defined, resulting in a simple, sensitive, and effective assay to sequence nearly full-length HIV-1 genomes from virion RNA.

## Materials and Methods

### Study Subjects

Plasma samples were obtained from the Molecular Diagnostics Laboratory at the University of Maryland Medical Center in Baltimore, MD, a CAP-accredited facility. Unidentified, archived samples were selected based on the VL, ranging from 1,000 to over 75,0000 copies/ml. VL testing was performed using the COBAS Amplicor HIV-1 Monitor Test, version 1.5. (Roche Molecular Systems Inc., Durham, NC).

### RNA Extraction

RNA was extracted from 500 µl HIV-1 positive plasma specimens using QIAamp Viral RNA Mini Assay (Qiagen, Valencia, CA). Virions were initially concentrated from the plasma or serum by centrifugation at 21,000×g for 75 min. All but 140 µl of the supernatant was removed before lysis buffer was added. Extracted RNA (60 µl) was either immediately reverse transcribed or stored at −80°C until use.

### Reverse Transcription

**Figure 1:**
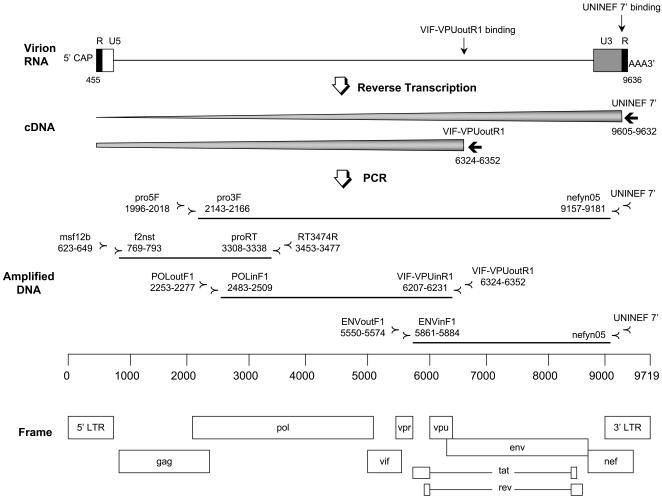
Nearly full-length RT-PCR method. Viral RNA was reverse-transcribed by priming with UNINEF 7′ or with VIF-VPUoutR1 using SuperScript^TM^ III RNase H^−^ RT. The locations of all the primers used for cDNA synthesis and nested PCR were depicted in this diagram with the map of the complete HIV-1 genome. Two different strategies were employed to amplify the nearly full-length genome: one amplified the 2.6-kb (*gag-pol*) and 7.0-kb (*pol-nef*) fragments with the overlap of 797-bp, and the other amplified three overlapping fragments of 2.6-kb (*gag-pol*), 3.7-kb (*pol-vpu*) and 3.3-kb (*env-nef*) with the 797-bp and 321-bp over lap regions, respectively.

The methods for RT- PCR are illustrated in Figure 1. Reverse transcription of the RNA was performed by priming with UNINEF7′ (Table 1) close to the 3′ end of the viral RNA or by VIF-VPUoutR1 (Table 1) in the *vpu* gene. The extracted RNA (3 µl) was reverse transcribed in a total volume of 20 µl with 500 µM dNTP, 2.5 µM primer, 1× RT buffer, 5 mM MgCl2, 10 mM DTT, 40 U RnaseOUT, and 400 U SuperScript^TM^ III RNase H^−^ RT (Invitrogen, Carlsbad, CA). The RNA, primer and dNTPs were first incubated at 65°C for 5 minutes, then the remaining reagents were added for cDNA synthesis at 50°C for 2 hours, followed by 85°C for 5 minutes. Then 2 U E. coli RNase H (Invitrogen, Carlsbad, CA) was added, and the reaction tubes were incubated at 37°C for 20 minutes followed by 70°C for 15 minutes.

**Table 1:** Primers for the amplification of nearly full-length HIV-1 genome.

Primer^a^	Position^b^	Sequence (5′→3′)	Length	Tm (°C)
msf12b (+)	623–649	AAATCTCTAGCAGTGGCGCCCGAACAG	27	76.04
f2nst (+)	769–793	GCGGAGGCTAGAAGGAGAGAGATGG	25	71.73
pro5F (+)	1996–2018	AGAAATTGCAGGGCCCCTAGGAA	23	71.04
pro3F (+)	2143–2166	AGANCAGAGCCAACAGCCCCACCA	24	75.44
POLoutF1 (+)	2253–2277	CCTCAAATCACTCTTTGGCARCGAC	25	70.36
POLinF1 (+)	2483–2509	AGGACCTACRCCTGTCAACATAATTGG	27	66.76
proRT (−)	3308–3338	TTTCCCCACTAACTTCTGTATGTCATTGACA	31	70.77
RT3474R (−)	3453–3477	GAATCTCTCTGTTTTCTGCCAGTTC	25	65.18
ENVoutF1 (+)	5550–5574	AGARGAYAGATGGAACAAGCCCCAG	25	68.83
ENVinF1 (+)	5861–5884	TGGAAGCATCCRGGAAGTCAGCCT	24	72.73
VIF-VPUinR1 (−)	6207–6231	CTCTCATTGCCACTGTCTTCTGCTC	25	69.28
VIF-VPUoutR1 (−)	6324–6352	GGTACCCCATAATAGACTGTRACCCACAA	29	68.49
nefyn05 (−)	9157–9181	GTGTGTAGTTCTGCCAATCAGGGAA	25	68.9
UNINEF 7′ (−)	9605–9632	GCACTCAAGGCAAGCTTTATTGAGGCTT	28	72.59

a (+) Sense primer, (−) Antisense primer.

b Positions according to HXB2 Numbering System.

### PCR Amplification

Two or three regions of the viral genome were independently amplified using nested PCR from the cDNA to contain a nearly full-length genome of HIV-1 (amplification primers listed in Figure 1 and Table 1). The two-amplicon strategy consisted of one amplicon of about 2.6-kb consisting of *gag* and part of *pol* (nts 769–3338 HXB-2 (Genbank Acc No: K03455)) and another amplicon of approximately 7.0-kb stretching from the 5′ end of *pol* to the middle of *nef* (nts 2143–9181 HXB-2). The reaction volume was 50 µl, containing 1× PCR buffer, 350 µM dNTP mixture, 0.4 µM of each primer and 5 U Expand Long Template PCR enzyme mixture (Roche Diagnostics, Indianapolis, IN). Cycling conditions were 94°C for 2 minutes and then 10 cycles of (94°C 10 s, 60°C 30 s, 68°C 3 min), then 20 cycles of (94°C 10 s, 55°C 30 s, 68°C 3 min) followed by 68°C for 10 minutes. For the second amplicon, cycling conditions were the same, except that the annealing temperature for first 10 cycles was 65°C and the incubation at 68°C in each cycle was for 8 minutes. A hot-start using DynaWax (Finnzymes, Espoo, Finland) was employed in each PCR, with two layers separated by wax. In the bottom layer were the dNTPs and primers while the top layer contained the PCR buffer, DNA polymerase and template cDNA. Limiting dilution of template into the first round was used to minimize the chances of producing mosaic molecules. The amplicon that could be obtained with the lowest amount of cDNA input was applied to the following sequencing step.

A three-amplicon strategy was used as needed. The first amplicon was the *gag*-*pol* amplicon described above, the second spanned *pol* to *vpu* (nts 2483–6231 HXB-2) and the third *env* to *nef* (nts 5861–9181 HXB-2) (Figure 1 and Table 1). The cycling conditions for these two regions were the same as the 2.6-kb *gag-pol* region, except that the incubation at 68°C in each cycle was for 4 minutes rather than 3 minutes.

### Sequencing

The PCR products for sequencing were filtered with Microcon YM-50 (Millipore Corporation, Bedford, MA, USA), and then the PCR products were directly sequenced using fluorescently labeled dideoxy chain terminators (BigDye® Terminator v3.1 Cycle Sequencing Assay, Applied Biosystems) and an automated 3130xl sequencer (Applied Biosystems). The primary set of sequencing primers used for each amplicon is listed in a supplemental material (Table S1). Sequencing reactions were repeated with alternate primers as needed to fill any gaps present to complete the sequencing of both DNA strands in each amplicon. The nearly full-length genome sequence was assembled by overlapping the sequences of the two or three amplicons and merging them into one sequence as long as the two were greater than 98% homologous.

## Results

### Optimization of Extraction Method

QIAamp viral RNA mini assay was selected after comparisons with other commercially available extraction assays for its reliability and efficacy. RNA extraction using a volume of 500 µl was useful to improve sensitivity for low VL samples. To show the standardized correlation between VLs and RT-PCR efficacies, all the samples listed in Table 2 were concentrated from the original volume of 500 µl.

**Table 2:** Comparative Sensitivities of HIV-1 Viral loads in Plasma samples for RT-PCR Detection of HIV-1 RNA.

	Viral Load (HIV-1 RNA copies/ml)				
	>750,000	100,000–200,000	10,000–100,000	2,000–10,000	1,000–2,000
	(% Efficiency^a^)	(% Efficiency^a^)	(% Efficiency^a^)	(% Efficiency^a^)	(% Efficiency^a^)
PCR Regions	n = 8	n = 8	n = 5	n = 4	n = 2
Env-Nef∼3.3 kb	8/8 (100%)	8/8 (100%)	4/5 (80%)	3/4 (75%)	0/2 (0%)
Pol-Vpu∼3.7 kb	8/8 (100%)	6/8 (75%)	2/5 (40%)	0/4 (0%)	0/2 (0%)
Gag-Pol∼2.6 kb	8/8 (100%)	6/8 (75%)	2/5 (40%)	1/4 (25%)	0/2 (0%)
Pol-Nef∼7.0 kb	6/8 (75%)	4/8 (50%)	2/5 (40%)	0/4 (0%)	0/2 (0%)

a Positive polymerase chain reaction product.

### Optimization of Reverse Transcription

Several different types of commercially available RT were compared, and SuperScript™ III RNase H^−^ RT had the highest yield. The incubation temperature for cDNA synthesis was 50°C, which was recommended by the manufacturer. The yield did not increase when temperature was raised to 53°C and 55°C.

The following three primers were compared for the reverse transcription: Oligo dT, random hexamer and the specific primer for HIV-1 genome, UNINEF 7′. Generally, priming with Oligo dT and UNINEF 7′ resulted in much better yields for synthesizing full-length cDNA than random hexamer, and UNINEF 7′ tended to have more consistent results than Oligo dT. Therefore, UNINEF 7′ was used for all the reverse transcription reactions to obtain the data for the further experiments.

Originally 200 U of SuperScript™ III RT was employed in a 21 µL reaction volume; however, the increased input of the enzyme to 400 U significantly improved the efficacy of RT-PCR. In the samples with the VL range of 10,000–200,000 copies/ml, an increase of 30% was observed for the 7.3 kb *pol-nef* region and 10% for the 2.6 kb *gag-pol* region.

### Primer design and optimization of PCR

The primers, listed in the legend to Table 1, were designed using the full-length genome alignment of diverse group M HIV-1 strains from the HIV sequence database [2]. The conserved sites surrounding or within the target region of the HIV-1 sequences were examined for optimal PCR primer sequences. The primers were designed to achieve annealing at a temperature of >55°C, and to avoid hairpins, self-priming, and primer-dimer formation. Primers were screened for specificity and evaluated to be able to amplify most of the strains within the diverse 9 subtypes of the group M HIV-1 using the Primalign HIV sequence database tool [12]. In addition, ambiguous bases within the primers were used to represent the genetic diversity present at the primer binding sites.

### Comparative Sensitivities of HIV-1 VLs for successful PCR amplification

The ability to successfully amplify and sequence HIV-1 RNA from plasma was dependent on VL. The frequency of positive amplifications of the four different fragments obtained in the 27 samples is shown in Table 2. The efficacy on the table is based on cDNA synthesis from the 3′end of viral RNA with primer, UNINEF 7′. In our initial strategy of amplifying two regions, a low efficiency of amplification of the 7.0-kb *pol-nef* fragment was observed in the samples with low VL. Therefore, the three-amplicon strategy was implemented for the samples that failed using the first approach. To measure the correlation between the VL and the efficacy of amplification, the PCR reactions were repeated at least 4 times with different dilutions to determine each as either positive or negative. The success rates for obtaining all amplicons for full-length were 100% in samples with a VL greater than 750,000 copies/ml, 75% in samples with a VL of 100,000–200,000 copies/ml, and 40% in samples with a VL of 10,000–100,000 copies/ml. Finally, there was a 76% success rate if the three-amplicon strategy were used for those with a VL of greater than 10,000 copies/ml.

Reverse transcription was also performed priming with VIF-VPUoutR1 for the samples with negative *vif-vpu* region amplification results. Approximately 60% of the previously negative samples were successfully amplified in the *pol-nef* and *gag-pol* region after priming with VIF-VPUoutR1, resulting in 90% efficiency for both regions for samples with a VL of greater than 10,000 copies/ml (data not shown).

### Analysis of Sequences of HIV-1 RNA

The reverse-transcription and PCR methods were verified by directly sequencing the nested PCR products amplified in all three regions in 15 samples. The amplified material present in strong bands obtained with PCR was sufficient for good quality sequences in direct automated fluorescent sequencing. All the amplicons were sequenced separately and assembled for the nearly full-length HIV-1 genome (sequence data will be published separately). The resulting sequences were around 8500-bp (nts 769–9181 HXB-2) and contained the nearly entire HIV-1 coding region, but lacked the 3′end of the *nef* gene and U3 and R of LTR regions (nts 9181–9636 HXB-2). All of the reading frames were open without apparent insertion, deletion, or rearrangement.

In the 2-amplicon and 3-amplicon strategies, there were regions of overlap between the adjacent amplicons. The overlapping regions of 797-bp and 321-bp were carefully analyzed to assemble the three fragments. Some overlapping regions displayed a small number of heterogeneous single-base substitutions, but the most common substitutions were G to A, which reflected the G to A hypermutation of one of the templates but not the other. To ensure that the sequence fragments were derived from the same genomic species, two criteria had to be met to represent a consensus sequence: the genetic distance had to be no greater than 2%, and the base differences had to be distributed evenly throughout the overlapping region. If these two criteria were met, a single sequence was generated; otherwise cDNA synthesis and PCR amplification were repeated.

## Discussion

The accuracy of direct sequencing of full-length virion RNA from HIV-1 relies on the ability to extract RNA from clinical materials, to conduct cDNA synthesis, and to amplify the cDNA successfully. The presence of an adequate number of intact RNA templates was essential for accurate and complete reverse transcription. An ultracentrifugation step to concentrate the viral particles before the extraction of RNA contributed to increased success especially for plasma samples with low VLs; a total of at least 250 virus copies (10,000 copies/ml×500 µl plasma÷60 µl RNA×3 µl used) had to be present for the procedure to be successful. Therefore the low abundance of viral RNA in some clinical samples remains a barrier to this application. However, most HIV-1 infected individuals have viral RNA titers that exceed 10,000 copies/ml in plasma if untreated or if they are on nonsuppressive antiretroviral therapy [13], [14], and thus this protocol can be employed for a broad range of plasma samples especially from infected individuals not under treatment, on suboptimal therapy, and in treatment failure.

Efficient cDNA synthesis is another important factor for successful RT-PCR. Increased efficiency of cDNA synthesis has been reported with a genetically engineered Moloney Murine Leukemia Virus (M-MLV) RT devoid of RNase H activity compared with unmodified M-MLV RT or AMV RT [15]. SuperScript^TM^ III RNase H^−^ RT is M-MLV with such introduced point mutations. The manufacturer (Invitrogen) recommends 200 U of RT for sufficient cDNA synthesis; however increasing the input of RT to 400 U significantly improved the amplification results. It is probably because a significant amount of thermal-sensitive RT is degraded during the incubation. Since the original 200 U input is more cost-effective than 400 U, we recommend the use of 200 U RT for samples with VL >750,000 copies/ml.

To synthesize full-length cDNA efficiently has been difficult to achieve presumably because the RT tends to be interrupted at regions of secondary structure in mRNA templates [16], [17], resulting in the generation of truncated cDNA. Even though the M-MLV RNase H^−^ enzyme appears to be the best at negotiating regions of secondary structure compared to other available *in vitro* RT [18], as the length of mRNA increases, the RT is more likely to encounter such regions, decreasing the chances of achieving the 5′end of template RNA. At 55°C some secondary structural elements begin to melt, and many existing protocols aim to perform reactions at increased temperatures with minimum interference with the activity of RT [19], [20]. However, the half-life of SuperScript^TM^ III RT significantly declines as temperature is raised: 220 min at 50°C and 30 min at 55°C. More importantly, elevated temperatures augment RNA breakdown; RNA is thermolabile and susceptible to divalent Mg^2+^-catalyzed degradation [18], [21], [22]. The RT requires divalent cations, and the reaction condition of MgCl_2_ with SuperScript III is 6 mM. It was reported that the rate of RNA breakdown is directly proportional to Mg^2+^ concentration between 1 and 10 mM at above 37°C, and is increased 1.75-fold for every 5°C increment in temperature increase above 40°C in the presence of 3 mM Mg^2+^ [21], [22]. Therefore, although high temperature contributes to resolving secondary structures of RNA, chances for a full-length mRNA template are much reduced. For those reasons, reverse transcription was carried out at 50°C in our protocol.

A high DNA recombination frequency during long RT-PCR has been previously reported [23], [24]. Recombination frequencies resulting from the combined procedure of long RT-PCR are especially higher than long PCR itself or RT-PCR of shorter molecules [23]–[26]. For accurate characterization of viral RNA genomes derived from HIV-1 infected plasma, it is necessary to minimize artifactual recombination between quasispecies during RT-PCR. High recombination frequencies are assumed to be related to the presence of partial cDNA that results from incomplete cDNA synthesis as well as partial RNA templates caused by degradation. According to the previous studies [23], [24], the following conditions were recommended in order to decrease recombination frequencies during long RT-PCR: the minimum quantity of input HIV-1 RNA, use of mixtures of DNA polymerase and proofreading enzymes, increased incubation time for cDNA synthesis, and increased extension time and a minimum number of cycles for PCR. The last two are associated with achieving complete strand synthesis to avoid PCR-mediated recombination. A 120 min RT incubation time and an extension time of 1.5–2 min/kb of template DNA were suggested as suitable to minimize recombination in most long RT-PCR [24]. We employed a 120 min RT incubation time in this protocol after multiple experiments, and since the excess extension time may compromise yield and specificity, the optimal PCR extension time was determined based on template lengths and extension rates of the DNA polymerase.

When the nearly full-length genome sequences were assembled, the overlapping regions displayed a small number of heterogeneous single-base substitutions. Most of them were probably because heterogeneous quasispecies exist in HIV-1 infected plasma samples. Any HIV sample contains a population of related but genetically diverse variants due to the high misincorporation rate of the HIV-1 RT [8], [27], [28]. Heterogeneous bases could also result from mutations created during the *in vitro* reverse transcription step; the M-MLV RT is error prone for incorporation of a wrong nucleotide because of the lack of 3′→5′ proofreading exonuclease activity [19], [29]–[31]; mutations created during the reverse transcription are carried on to the final products to be analyzed, which may interfere with accurate analysis of viral RNA. The actual empirical error rates of the SuperScript^TM^ III RT as used in our assay are unknown. The G to A single-base substitutions were the most commonly observed among the heterogeneous bases in the overlapping regions. Recent reports showed that a cytosine deaminase APOBEC3G (aplolipoprotein B mRNA-editing enzyme, catalytic polypeptide-like 3G), which is packaged in HIV-1 virions, induces G to A hypermutation to a nascent reverse transcript of HIV-1, which contributes in part to the innate antiviral activity [32]–[35]. Even though the sequence is not from an infectious virus, the hypermutated sequences can still be used for molecular subtyping.

The evidence of dual infections and recombination between subtypes[36], [37] suggest that amplification of fewer fragments is desirable; while the two-amplicon strategy had an advantage of increasing the possibility of amplifying the single species of HIV-1, the three-amplicon strategy was more sensitive for amplification of samples with low VLs. In a study with SuperScript™ II MMLV RT, it was indicated that cDNA synthesis continued to 6-kb and decreased afterwards [38]. The 5′ end of an mRNA is generally represented to a lesser extent than the 3′ end in cDNA. This is in accordance with the increased sensitivity of amplification after cDNA synthesis with primer, VIF-VPUoutR1, compared to UNINEF 7′ in our study. The limiting dilution method for PCR was used because it is most likely to pick the predominant strain of the viral population. The relatively long overlap regions (797- and 321-bp) between the amplicons increase the likelihood of identifying and constructing full-length amplicons of the single molecular species.

Previous study has showed a method for the amplification and sequencing of nearly full-length HIV-1 genomes from RNA[39]. However, their primers were designed based on the sequences in their South African cohorts and amplify HIV-1 subtype C preferentially. The primers presented in this paper were designed to be able to pick up most of the strains from the group M HIV-1 available in the HIV sequence database [2]. Additionally their PCR products need to be cloned with a commercially available cloning kit in comparison with our protocol with direct-PCR sequencing. Addition of a cloning step is more time-consuming and can introduce the possibility of bias. Therefore, the methods described here provide a more comprehensive protocol of sequencing nearly full-length HIV-1 RNA compared to currently described methods.

Since this protocol is still not applicable to a low VL range of HIV-1 infected plasma and serum specimens, further optimization of the protocol in the future will be necessary. Recent publications reported a number of reagents that are supposed to facilitate the disruption of RNA secondary structure and to improve an ability to achieve full-length cDNA synthesis, including EDTA to eliminate free Mg^2+^, addition of DMSO, RNA chaperone, formamide and glycerol, and pretreatment of the RNA with methylmercury hydrozide [18], [20], [22], [40], [41]. Recently it has also been documented that an incorporation of a proofreading function to RT significantly increased the full-length content of cDNA up to 15-kb [42]. However, the effectiveness of these conditions has never been tested in controlled experiments harboring defined structural features capable of blocking RT activity, therefore supplementary testing will be required before implementing them.

In summary, a new protocol for sequencing nearly full-length HIV-1 RNA genomes directly form plasma using long RT-PCR was described in this study. The success of the procedure was sensitive to VL but was routinely successful for VL greater than 10,000 copies/ml. The method used in this study overcomes some of the practical difficulties found when trying to sequence nearly full-genomic HIV-1 from virion RNA. Its importance is two-fold: first, it confers the ability to sequence the HIV-1 genomes of newly circulating viruses; second, it can be used to study the molecular epidemiology of HIV-1 in regions of the world where only serum or plasma is available.

## Supporting Information

Table S1 (0.14 MB DOC)
