# Supplementary material for: Protocol for Nearly Full-Length Sequencing of HIV-1 RNA from Plasma
Source: PLoS One. 2008 Jan 9;3(1):e1420. doi: 10.1371/journal.pone.0001420 (PMC2170516; doi:10.1371/journal.pone.0001420)
Supplement: Table S1 — (0.14 MB DOC) [file pone.0001420.s001.doc]

| **Table S1.** Primary set of primers for sequencing nearly full-length HIV-1 genome | | | |
| --- | --- | --- | --- |
| **Amplicon Region** | | **Primer** | **Sequence (5' -> 3')** |
| Gag-Pol |  | F2NST | GCGGAGGCTAGAAGGAGAGAGATGG |
| Gag-Pol |  | DD | GTATGGGCAAGCAGGGAGCTAGAA |
| Gag-Pol |  | JL19 | CTTCTATTACTTTTACCCATGC |
| Gag-Pol |  | JL17 | CATTCTGCAGCTTCCTCATTGAT |
| Gag-Pol |  | HH | ATGAGGAAGCTGCAGAATGGG |
| Gag-Pol |  | II | ATAATCCACCTATCCCAGTAGGAGAAAT |
| Gag-Pol |  | POLCLO1- | GAGAGACAGGCTAATTTTTTAGGGAA |
| Gag-Pol |  | SP2AS | GGTGGGGCTGTTGGCTCTG |
| Gag-Pol |  | BJPOL1 | ACAGGAGCAGATGATACAGTA |
| Gag-Pol |  | SP3AS | CCTCCAATTCCCCCTATCATTTTTGG |
| Gag-Pol |  | SP4AS | AGTATTGTATGGATTTTCAGGCCC |
| Gag-Pol |  | AZT9 | TGGATGTGGGTGATGCATA |
| Gag-Pol |  | SP5S | GGATTAGATATCAGTACAATGTGC |
| Gag-Pol |  | AZT5 | TCAGATCCTACATACAAATCATCCATGTATTG |
| Gag-Pol |  | AZT4 | TATAGGCTGTACTGTCCATTT |
| Gag-Pol |  | proRT | TTTCCCCACTAACTTCTGTATGTCATTGACA |
| Pol-Vpu |  | POLinF1 | AGGACCTACRCCTGTCAACATAATTGG |
| Pol-Vpu |  | AZT3 | CCAGGAATGGATGGACCAA |
| Pol-Vpu |  | SP4S | GGGCCTGAAAATCCATACAATACT |
| Pol-Vpu |  | SP4AS | AGTATTGTATGGATTTTCAGGCCC |
| Pol-Vpu |  | AZT9 | TGGATGTGGGTGATGCATA |
| Pol-Vpu |  | POLC- | CTAGGTATGGTAAATGCAGTATA |
| Pol-Vpu |  | AZT10 | CCTACATACAAATCATCCATGTATTG |
| Pol-Vpu |  | AZT6 | CAATACATGGATGATTTGTATGTAGG |
| Pol-Vpu |  | POLP | GGATGGGATATGAACTCCATCC |
| Pol-Vpu |  | POLEE- | TGTATGTCATTGACAGTCCAGCTG |
| Pol-Vpu |  | DGPOLF7 | GGAATATATTATGACCCATCAAAAGAC |
| Pol-Vpu |  | POLSEQ3 | GATATGWCCACTGGTCTTGCCC |
| Pol-Vpu |  | DGPOL3R | GTATTGACAAACTCCCAGTCAGGAAT |
| Pol-Vpu |  | POLU | ACTTTCTATGTAGATGGGGCAGC |
| Pol-Vpu |  | POLI | GAGCAGTTAATAAAAAAGGAA |
| Pol-Vpu |  | POLI- | TTTGTGTGCTGGTACCCATGCCAG |
| Pol-Vpu |  | POLJ | GAAGCCATGCATGGACAAGTAGA |
| Pol-Vpu |  | POLT- | GCAGTCTACTTGTCCATGCATGGC |
| Pol-Vpu |  | POLK | ACGGTTAAGGCCGCCTGTTGGTGG |
| Pol-Vpu |  | SP1AS | GGATGAATACTGCCATTTGTACTGC |
| Pol-Vpu |  | POLSEQ2 | CGGGTTTATTACAGGGACAGC |
| Pol-Vpu |  | DGPOL2R | CACTATTGTCTTGTATTACTAC |
| Pol-Vpu |  | ACC1 | TTCAGAAGTATACATCCCACTAGG |
| Pol-Vpu |  | ACC2 | AGGGTCTACTTGTGTGYTATAT |
| Pol-Vpu |  | VIFB | ATATAGCACACAAGTAGACCCT |
| Pol-Vpu |  | VIFC | GAYAAAGCCACCTTTGCCTAGTGTT |
| Pol-Vpu |  | ACC6 | GCTTGTTCCATCTRTCYTCTGTYAG |
| Pol-Vpu |  | ACC5 | TGAAACTTAYGGGGATACTTGG |
| Pol-Vpu |  | ACC4 | CCAAGTATCCCCRTAAGTTTCA |
| Pol-Vpu |  | ED3 | TTAGGCATCTCCTATGGCAGGAAGAAGCGG |
| Pol-Vpu |  | ACC8R | TCTCCGCTTCTTCCTGCCATAG |
| Pol-Vpu |  | VIF-VPUinR1 | CTCTCATTGCCACTGTCTTCTGCTC |
| Env-Nef |  | ENVinF1 | TGGAAGCATCCRGGAAGTCAGCCT |
| Env-Nef |  | ED3 | TTAGGCATCTCCTATGGCAGGAAGAAGCGG |
| Env-Nef |  | GP1205- | AGAGCAGAAGACAGTGGCAATGA |
| Env-Nef |  | ES33 | CATTGCCACTGTCTTCTGCTC |
| Env-Nef |  | Z1F | TGGGTCACAGTCTATTATGGGGTACCT |
| Env-Nef |  | JL99 | TTTAGCATCTGATGCACAAAATAG |
| Env-Nef |  | ENVSEQ22 | GTGTACCCACAGACCCCAGCCCACAAG |
| Env-Nef |  | ZFF | GGGATCAAAGCCTAAAGCCATGTGTAA |
| Env-Nef |  | 793SEQ1 | AACACCTCAGTCATTACACAGGCC |
| Env-Nef |  | AENVSEQ4 | CAAGCTTGTGTAATGGCTGAGG |
| Env-Nef |  | E16 | CCAATTCCCATACATTATTGTG |
| Env-Nef |  | TUE3 | TCCTTCTGCTAGACTGCCATTTA |
| Env-Nef |  | E15 | GTAGAAATTAATTGTACAAGACCC |
| Env-Nef |  | OFM54 | TTTAATTGTGGAGGGGAATTTTTCT |
| Env-Nef |  | JL98 | AGAAAAATTCCCCTCCACAATTAA |
| Env-Nef |  | E13 | ACAAATTATAAACATGTGGCAGG |
| Env-Nef |  | JL102 | GATGGGAGGGGCATACAT |
| Env-Nef |  | EDS8 | CACTTCTCCAATTGTCCCTCA |
| Env-Nef |  | JL109 | GTGAATTATATAAATATAAAGTAG |
| Env-Nef |  | TUG | GTCTGGTATAGTGCAACAGCA |
| Env-Nef |  | TUH | GCCCCAGACTGTGAGTTGCAACAGATG |
| Env-Nef |  | FM116 | CAGAGATTTATTACTCCAACTA |
| Env-Nef |  | ZLF | GGGATAACATGACCTGGATGCAGTGGG |
| Env-Nef |  | JL104 | GGAGGCTTGATAGGTTTAAGAATA |
| Env-Nef |  | ENVSEQ6 | CCTGCCTAACTCTATTCAC |
| Env-Nef |  | JL106 | TTCAGCTACCACCGCTTGAGAGACT |
| Env-Nef |  | E8 | CTCTCTCTCCACCTTCTTCTTC |
| Env-Nef |  | NEF7 | TAAGATGGGTGGCAAGTGGTCCAAAA |
| Env-Nef |  | JL71 | TTTTGACCACTTGCCACCCAT |
| Env-Nef |  | NEF6 | AGCAGCAGATGGGGTGGGAGCAG |
| Env-Nef |  | JL89 | TCCAGTCCCCCCTTTTCTTTTAAAAA |
| Env-Nef |  | NEFYN05 | GTGTGTAGTTCTGCCAATCAGGGAA |
